# Calibration of BRDF Based on the Field Goniometer System Using a UAV Multispectral Camera

**DOI:** 10.3390/s22197476

**Published:** 2022-10-02

**Authors:** Minji Kim, Cheonggil Jin, Sejin Lee, Kyoung-Min Kim, Joongbin Lim, Chuluong Choi

**Affiliations:** 1 Division of Earth Environmental System Science (Major of Spatial Information Engineering), Pukyong National University, Busan 49513, Korea; 2 Forest ICT Research Center, National Institute of Forest Science, Seoul 02455, Korea

**Keywords:** bidirectional reflectance distribution function (BRDF), unmanned aerial vehicle (UAV), adjacency effect, multispectral camera, irradiance, reflectance, calibrated reference tarp (CRT), remote cosine receptor (RCR), anisotropy factor (ANIF)

## Abstract

The bidirectional reflectance distribution function (BRDF) is important for estimating the physical properties of a surface in remote sensing. In the laboratory, the BRDF can be estimated quickly and accurately using a goniometer, but it is very difficult to operate in the field. The purpose of this study was to evaluate whether estimating the BRDF with reasonable accuracy using an unmanned aerial vehicle (UAV) with a multispectral camera is possible in the field. Hemispherical reflectance was created from images taken using an UAV multispectral camera. The ground targets were four calibrated reference tarps (CRTs) of different reflectance, and the UAV was operated five times. Down-welling irradiance for reflectance calculation was measured in two ways: a sunlight sensor was mounted on a UAV, and a spectroradiometer with a remote cosine receptor (RCR) was installed on the ground. The BRDF was assessed through the anisotropy factor (ANIF) of the CRT reflectance derived from the collected data. As a result, the irradiance data for the reflectance calculation were more effective from the spectroradiometer with RCR on the ground than from the sunlight sensor mounted on an UAV. Furthermore, the high reflectance CRTs, ANIF, and BRDF had similar results. Therefore, when analyzing the BRDF, the effectiveness can be guaranteed when the reflectance of the target is over 21~46%, because a low reflectance tendency differs due to the adjacency effect. In addition, weather affects irradiance, so it is more effective to conduct fieldwork in clear weather.

## 1. Introduction

As most of the Earth’s ground surfaces are non-anisotropic, incident solar light, regardless of the angle, does not scatter energy in any direction [1]. That is, since the surface of the Earth is non-Lambertian, incident energy from different angles is then reflected from different angles. Therefore, a goniometer is used to evaluate and determine the spectral direction reflectance characteristics of natural or artificial targets [2].

The bidirectional reflection distribution function (BRDF) is a reflectance characteristic of a specific area, and it describes a phenomenon in which a ray of incident light is scattered from one given angle of a hemisphere [3,4]. By estimating the BRDF, it is possible to gauge the surface structure and physical properties of the specific target in remote sensing [5]. In addition, reflectance is a material property that maintains a constant value and the properties of incident light, unlike radiance and irradiance. Therefore, reflectance is used to differentiate the spectral measurement results of a target from its reflection characteristics in remote sensing [6]. The BRDF is a method capable of representing the characteristics of the reflectance of the target surface, thus demonstrating that the reflectance varies when the viewing angle changes according to the characteristics of the target surface and irradiance [7].

In other research, a hemispherical goniometer has mainly been used to estimate the BRDF. Among the goniometers mainly used in field work for BRDF estimation are the Portable Apparatus for Rapid Acquisition of Bidirectional Observation of the Land and Atmosphere (PARABOLA) [8,9], University of Lethbridge Goniometer System (ULGS-2) [10,11], Gonio RAdiometric Spectrometer System (GRASS) [12,13,14,15], and Goniometer for Outdoor Portable Hyperspectral Earth Reflectance (GOPHER) [16,17,18]. These goniometers are not portable because of their large size, and some can only acquire the surface data for a quarter of the hemisphere. Because ground radiance data change with incident energy, they must be acquired quickly. In this respect, a large goniometer may not be efficient. The traditional field goniometer weight is approximately 500 pounds, taking an estimated 90 min to assemble. The technical cost is more than USD 125,000 [19].

A laboratory robotic goniometer is often used [20,21,22]. A robotic goniometer is expensive and can measure a target of 20–30 cm or less in a laboratory with high-precision data within 1–10 min. However, when operating in the field, it is very difficult to use a robotic goniometer without electricity, and it is even more difficult to use it for measurements of large targets (over 10 m).

A multispectral camera for BRDF estimation has also been used [23,24,25,26]. However, there are a few cases of BRDF estimations with an unmanned aerial vehicle (UAV) for hemispherical flights such as goniometers. This study presents a method for acquiring multispectral images in a more portable manner using a UAV goniometer for BRDF estimation. Afterward, the reflectance of a multi-VAA (vehicle azimuth angle) and a single-VZA (vehicle zenith angle) was analyzed. An experiment was conducted at the same nadir altitudes, and during this time, there were specific limitations within which it was unfeasible to observe when the target exceeded the instant field of view (IFOV). The other limitation was that the results were vastly affected by the vignetting effect of the UAV camera and the cloudy weather conditions.

A hemispherical flight was performed using an auto stable gimbal that adjusted the VAA and the VZA, and after programming a detailed vignette correction process to solve the vignetting problem, only the center of the image with the least vignette effect was utilized for analysis.

Therefore, in this study, the BRDF in the bands of B (450 nm), G (560 nm), R (650 nm), RE (730 nm), and NIR (840 nm) was estimated using a UAV equipped with a multispectral sensor with an auto-stabilized gimbal. In order to minimize the change in incident solar energy, the flight time was shortened as much as possible, and an auto-stabilized gimbal was used for accurate VAA and VZA control.

A total of 195 points were placed in the flight path for the BRDF estimation of a specific band. One observation period was 11–11.5 min, which was more than three times the time efficiency of the other studies.

The UAV used in this study was equipped with a sunlight sensor. The sunlight sensor measured the irradiance as a DN value when the UAV took an image. In order to compare the irradiance values measured by different methods, irradiance was measured two different ways: using the sunlight sensor on the UAV or the ASD (Malvern Panalytical Inc., Westborough, MA, USA) with a remote cosine acceptor (RCR) installed on the ground. Accordingly, the accuracy of the BRDF estimation was analyzed for each of the four calibrated reference tarps (CRTs) with the different reflectance values used in the fieldwork and for each band constituting a multispectral sensor.

## 2. Materials and Methods

### 2.1. The Research Material

**Figure 1:**
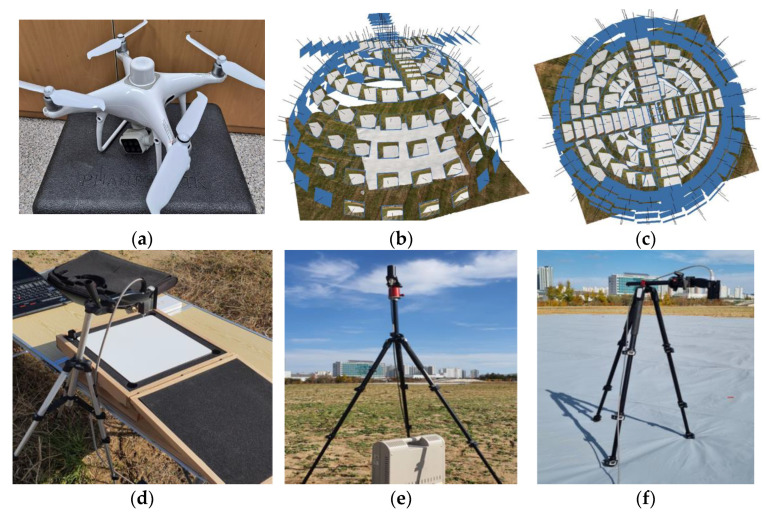
Hemispherical flight for BRDF and fieldwork: (**a**) P4M with real-time kinematic (RTK); (**b**) side view of UAV flight pattern for BRDF with RTK; (**c**) top view of UAV flight pattern for BRDF with RTK; (**d**) ASD No. 2 radiance measurement using white reference (WR); (**e**) ASD No. 3 irradiance measurement using remote cosine receptor (RCR); and (**f**) ASD No. 1 CRT measurement using ASD FieldSpec (Malvern Panalytical Inc., Westborough, MA, USA).

The field work was held on 29 October 2021 at the Department of Agricultural Engineering, National Institute of Agriculture Science road test field in Jeonju, South Korea. This study used a multispectral camera equipped with a P4 Multispectral (P4M), three ASD FieldSpec spectrometers, and four CRTs. The ASD FieldSpec spectrometer for ground observation was labeled ASD No.1 on the CRT, as shown in Figure 1f, ASD No. 2 for radiance with a Spectralon white reference panel (WR), as shown in Figure 1d, and ASD No. 3 for irradiance with RCR, as shown in Figure 1e.

For the CRT, 15 × 15 m targets with a constant reflectance of 3%, 20%, 31%, and 46% were used. The CRT had a flat reflectance response from solar irradiance at the visible band (VIS), RedEdge band (RE), and near-infrared band (NIR) on the ground. In addition, these CRTs were used as a standard reflectance for a multispectral camera.

**Figure 2:**
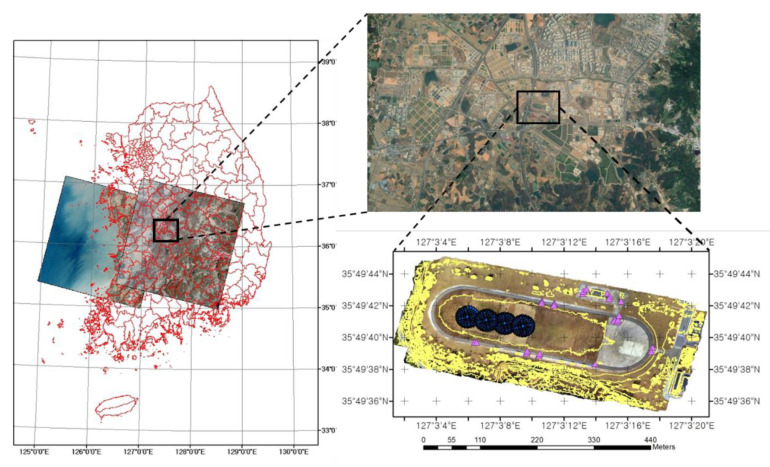
The location of the Jeonju calibration site.

The weather information was provided by the Korea Meteorological Administration, and the weather conditions (temperature, humidity, wind speed, and sea level pressure) for the multispectral data with P4M were 9.4~20.9 °C, 37~78%, 0.1~1.7 m/s, 1023.5~1026.5 hPa, and slightly cloudy, as illustrated in Figure 2, in the four CRTs at the fieldwork site.

The cloud type was altostratus cirrus at 5~13 km from the mean sea level (MSL). The cloud cover of this cirrus was partial (10~20% at 9 h, 10 h, and 12 h) and medium (50~60% at 8 h, 11 h, and 13 h) at the fieldwork site. However, cirrus was not listed as a heavy cloud.

The flight height of the UAV goniometer was 6.8 m (VZA 70°) to 20 m (VZA 0°(nadir)) from the ground, as shown in Figure 1b,c. The VZA and VAA interval for the BRDF and check flight is as shown in Table 1. The BRDF flight total images were 151 per band. The check images for the accuracy assessment image were 44 per band. The reflectance of each band was used for calibration and verification according to the manufacturer’s recommendation equation and procedure [27].

The P4M gathered precise data with real-time kinematic (RTK) for a positional datum (X, Y, Z) and a high-precision controllable camera gimbal system (roll, pitch, yaw). The positional accuracy was 1.5 cm + 1 ppm (vertical), 1 cm + 1 ppm (horizontal) [28]. The ground sample distance (GSD) was approximately 7.94 cm/pixel (F.H./18.9 cm).

In addition, this system had six cameras including one VIS color sensor and five multispectral (B (450 ± 16 nm), G (560 ± 16 nm), R (650 ± 16 nm), RE (730 ± 16 nm), and NIR (840 ± 26 nm)) monochrome sensors. The pixel size, IFOV, aperture, and focal length of each camera were 1600 × 1300 (4:3.25, 2.08 MP), 48.5°, F2.2, and 5.7 mm (40 mm at 35 mm), respectively. The camera shutter system was an electronic global shutter, and the shutter speed was 1/100~1/20,000 (for VIS) and 1/100~1/10,000 (for the multispectral camera).

### 2.2. Research Methodology

**Figure 3:**
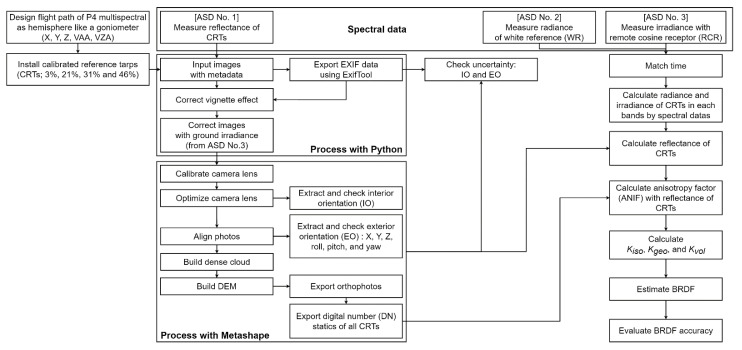
A flow chart of this study.

The research flowchart is shown in Figure 3. This study was divided into four separate stages. The first step was the CRT installation process and the design of the flight course. We structured a hemispherical flight center position with the radius in the flight course by creating a macro program using MS Office Excel; the camera position (X, Y, Z) was automatically calculated and converted to a KML file with a freeware program, and we utilized DJI Inc. (Shenzhen, China) GS PRO for the camera position (X, Y, Z) [29]. In addition, we manually entered the VZA, VAA, and roll.

The second step was to take images with a hemispherical flight for BRDF, and the radiance and irradiance were observed on the ground with a spectrometer. The flight course was a constant interval, as per a goniometer. The flight targets were the CRT 3%, 20%, 31%, and 46%. After the navigation obstacle check, the main flights were from 10:00 to 12:30 for each CRT. One time was noted for the CRT 3%, 20%, and 31% and two times for the 46% CRT, as shown in Table 2. There were 5850 BRDF flight photos and accuracy check images (195 (BRDF: 150, nadir: 1, check: 44) × 6 (multispectral: 5, color: 1) × 5 (flight)). Each of the flight times was recorded as approximately 11 min. A total of 1170 photos were captured on each flight. In addition, we used 4875 excluding the color VIS band.

The third step consisted of preprocessing the obtained photos through the Python program. For the preprocessing of images, we required the image metadata including the irradiance and radiance gathered from the aforementioned fieldwork. The metadata of the images were extracted using the ExifTool [30] and they included the flight course and the attitude of the P4M. 

There were two types of irradiance observations: the observation in the air (P4M) and the ground observation (ASD No. 3 with RCR), and the radiance was observed by ASD No. 2 with WR. In this study, the data from ASD No. 3 were used for preprocessing. Therefore, in this study, the vignetting effect and reflectance with irradiance correction was programmed using Python. 

The fourth step comprised aerial photo image processing for orthophotos employing Agisoft Inc. (Petersburg, Russia) Metashape [31]. Over 1000 photos were processed each time, thus requiring long periods of time for the existing aerial photographs. Therefore, in this study, we conducted parallel computing using Metashape with eight computers. Metashape was used for lens distortion compensation and orthorectification.

In the study, calculations were conducted using Metashape with the ground control point (GCP). The interior, relative, and absolute orientation was performed using the GCP and RTK of the UAV global navigation satellite system (GNSS). The digital elevation model (DEM) and orthophotos were produced based on the image using Metashape. In addition, we attached a subset image (512 × 512 pixels) in the center of the corrected vignette orthophoto for BRDF model fitting. A CRT was calculated using the data of ASD No. 1 on the ground with CRT and ASD No. 2 with a Spectralon WR, while the anisotropy factor (ANIF) was calculated using a nadir image (VZA and VAA = 0°) in the center of each hemispheric and reflectance based on the orthophoto reflectance of each VZA and VAA for BRDF fitting. Based on 151 orthophotos, the BRDF was calculated for each flight course and band to calculate the isometric ($k_{iso})$, geometric $(k_{geo}$), and volumetric $\left(k_{vol}\right)$ factor. The accuracy of the BRDF was calculated by using 44 check orthophotos.

### 2.3. The Reflectance, Bidirectional Reflectance Distribution Function (BRDF) Calculation, and Anisotropy Factor (ANIF) Calculated from P4 Multispectral(P4M)

We calculated the reflectance values using P4M images and irradiance using P4M sunlight sensors in the air and ASD on the ground. The general equation to calculate the reflectance ($\rho_{p4m\_ref})$ is as follows:

(1) $$R_{p4m\;}=\frac{L_{refleted}}{L_{incident}}=L_{p4m}\times \frac{p_{sensor\;gain}}{p_{irradiance}}\cdot D_{p4m}$$

where $L_{p4m}\;,\;L_{reflected},\mathrm{and}\;L_{incident}$ are the DN values from the P4M, the reflected radiance, and the incident irradiance of each band, respectively. Incident energy was calculated by irradiance from the sunlight sensor $\left(p_{irradiance}\right)$, and the $p_{sensor\;gain}$ was derived from the metadata. The removed vignette DN $\left(L_{p4m}\right)$ is a P4M image multiplied by $p_{sensor\;gain}$. $D_{p4m}$ is the downwelling irradiance correction ratio factor for each P4M image by ground observation.

$L_{p4m}$ was calculated by a vignetting correction parameter (v), shutter speed ($t_{e})$, and sensor gain (g) in the exchangeable image file format (EXIF) [27].

When the reflectance was measured from $\theta_{s}$ (sun zenith angle (SZA)), $\Phi_{S}\;$(sun azimuth angle (SAA)), $\theta_{v}\;$(VZA), and $\Phi_{v}\;$(VAA), the BRDF was resolved [32], and the distribution of the bidirectional reflectance of the object was determined according to the external lighting environment. Therefore, the BRDF was possible to predict with the use of ANIF by the goniometer [33].

The BRDF model has a similar fitting capability to the better kernel in the case of high SZA and VZA [34]. Therefore, in this study, the geometric scattering ($f_{geo})$ and volumetric scattering ($f_{vol})$ used the Li–Transit–Reciprocal [35] and the Ross–Thick–Maignan kernel [36]. The SZA, SAA, VZA, and VAA in the Ross–Thick BRDF model are calculated as shown in Equation (2):

(2) $$\rho \left(\theta_{v},\theta_{s},\varphi \right)=k_{iso}+k_{vol}\times f_{vol}\left(\theta_{v},\theta_{s},\varphi \right)+k_{geo}\times f_{geo}\left(\theta_{v},\theta_{s},\varphi \right);\;\;\left(\varphi =abs(\Phi_{s}-\Phi_{v}\right))\;$$

where $\rho ,\rho_{0},\varphi$ are the overlaying areas between the vehicle and the shadows of the Sun. The phase angle between SAA $(\Phi_{s}$) and VAA $(\Phi_{v}$) is the local relative azimuth angle (RAA).

The UAV multispectral images were numerous. For efficient image processing, each photo by VZA and VAA had different viewpoints and images in the orthophotos. 

The optimal number was 151, determined for each band and flight. The BRDF is based on isotropic $\left(k_{iso}\right)$, volumetric $\left(k_{vol},\;f_{vol}\right)$, and geometric $\left(k_{geo},\;f_{geo}\right)$ scattering effect parameters [37].

The ‘K’ ($k_{iso}$, $k_{vol}$ and $k_{geo}$) coefficients for the band and flight can be established using the least squares method, which is shown in Equations (3) and (4).

(3) $$F=\left[\begin{array}{ccc} 1 & f^{1}{}_{vol} & f^{1}{}_{geo}\\ \vdots & \vdots & \vdots \\ 1 & f^{n}{}_{vol} & f^{n}{}_{vol} \end{array}\right],K=\lfloor \begin{array}{c} k_{iso}\\ k_{vol}\\ k_{geo} \end{array}\rfloor ,\;\rho =\left[\begin{array}{c} R_{1}\\ \vdots \\ R_{n} \end{array}\right]\;\;F\cdot K=R,\;\;\Rightarrow \;\;\;K={\left[\begin{array}{cc} F^{T} & F \end{array}\right]}^{-1}\times F^{T}\times R$$

ANIF is a ratio index between any directional reflectance ($\;R_{b}\left(\theta_{s},\theta_{v},\Phi_{s},\Phi_{v}\right))$ and the nadir (VZA, VAA = 0°) reflectance ($R_{nadir}\left(\theta_{s},0,\Phi_{s},\;0\right)$). Furthermore, it is related to the corrected BRDF of the multispectral image [38], and the ANIF is the nadir corrected reflectance factor for the BRDF using Equation (4). That is, $R_{nadir}\left(\theta_{s},0,\Phi_{s},0\right)$ is the corrected apparent bottom of atmosphere (BOA) reflectance by the BRDF at nadir.

(4) $$ANIF\left(\theta_{s},\theta_{v},\Phi_{s},\Phi_{v}\right)=\frac{R_{b}\left(\theta_{s},\theta_{v},\Phi_{s},\Phi_{v}\right)}{\;R_{nadir}\left(\theta_{s},0,\Phi_{s},\;0\right)}\;$$

## 3. Results

### 3.1. The Assessment of Irradiance and Reflectance

The P4M has a sunlight sensor that records the incident energy signal values, and the irradiance data can be extracted from metadata using ExifTool. On the ground, the irradiance was measured by the ASD Inc. spectrometer FieldSpec with RCR. The RCR can measure the full hemispherical irradiance as well as corresponding reflected radiance.

**Figure 4:**
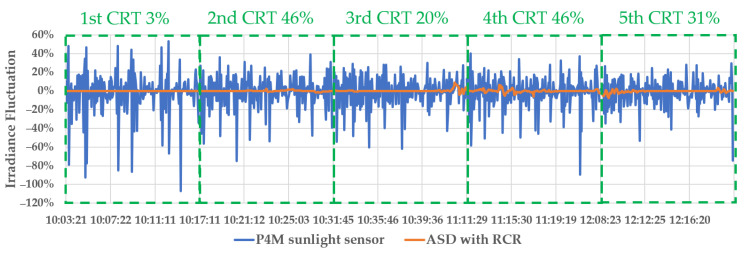
The irradiance fluctuation rate in air and ground.

The irradiance was measured on the ground and in the air five times in total. As a result, in Figure 4, the blue lines are the fluctuation rate of irradiance in the air in each flight, and the red lines are the fluctuation rate of irradiance on the ground in each flight, where the fluctuation rate is $\left({\mathrm{Irradiance}}_{n+1}-{\mathrm{Irradiance}}_{n}\right)/{\mathrm{Irradiance}}_{n}$.

The irradiance fluctuation on the ground had an almost constant value in clear weather. However, the irradiance measured by the P4M sunlight sensor was unstable due to a change in the vehicle attitude. The ground irradiance in Figure 4 shows a sine graph at each equivalent VZA. Because of changes in the VAA and VZA, this irradiance in the air (P4M) could not be stabilized and had a large fluctuation.

In Table 3, in the 1st CRT 3%, the CRT reflectance was 1.2%~2.2% in nadir. The check image reflectance was 1.7~2.4 ± 0.2~0.5%.

In CRT 20%, 31%, and 46%, the reflectance was 10.8%~19.1% 17.4~25.9%, 42.4~46.6% (2nd), respectively, and 44.4~49.7% (4th) in nadir. The check image reflectance was 11.7~19.4 ± 1.1~1.5%, 17.6~26.4 ± 0.9~2.0%, 43.4~46.9 ± 1.5~3.6% (2nd) and 44.8~50.8 ± 1.4~3.6% (4th).

The calculated reflectance differences in the nadir and check images were 0.2~1.1%.

Table 4 describes the standard deviation of the reflectance in the image before and after correction by the ground irradiance, with the reflectance derived through simulation using the ‘K’ coefficient value. According to the results of the 1st CRT 3%, the reflectance before and after correction in the BRDF and the check images was little changed by the clear weather conditions.

In the BRDF image, the CRT 20%, 31%, and 46%, the reflectance before and after correction in the BRDF was a little improved at 0.1~0.2%, 0.8~1.8%, and 1.0~5.9%, respectively. In the check image, the reflectance before and after correction was improved: −1.5~0.9%, 0.2~0.7%, and 0.8~3.3% excluding RE and NIR.

**Figure 5:**
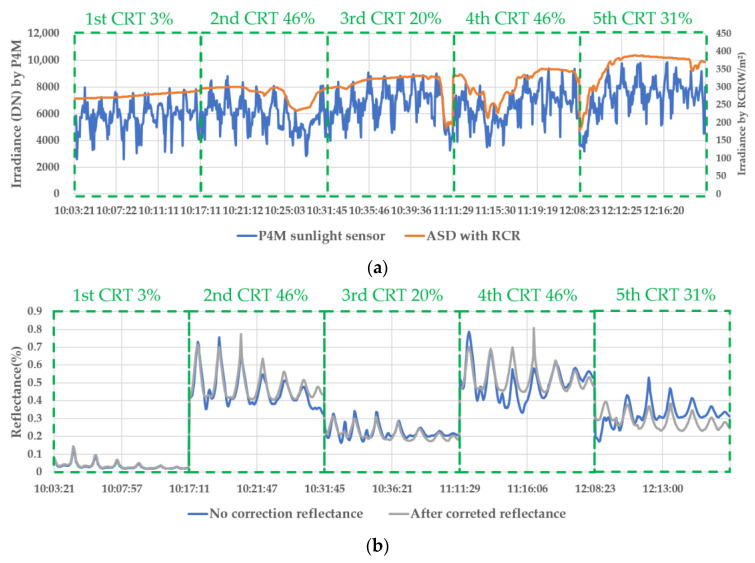
Irradiance and BRDF by correction type. (**a**) In air (P4M) and on ground irradiance (ASD with RCR), (**b**) BRDF by correction type.

During the 2nd CRT 46% and 4th CRT 46%, unstable irradiance occurred due to cloudy weather such as that shown in Figure 5a. The check image at the same flight height had fewer errors than the BRDF type image. Therefore, in an unstable irradiance and cloudy situation, we concluded that it will be greatly affected by the ground observation irradiance.

In Table 5, the 1st CRT 3%, the orthophotos of the CRT 3% were preprocessed for reflectance. The center CRT 3% reflectance was analyzed for uniformity and was approximately ±0.02~0.04% in the check image and ±0.02~0.05% in the BRDF image.

In CRT 20%, 31%, and 46%, uncertainty was approximately ±0.36~0.60%, ±0.81~1.64%, ±1.84~3.31% (2nd), and ±1.61~3.13% (4th) in check image and ±0.36~0.64%, ±0.74~1.58%, ±1.84~3.50% (2nd), and ±1.61~3.08% (4th) in the BRDF image.

The uncertainty increased from the blue band to the NIR band. CRT 46% was installed in a place with drainage channels and furrows, rather than on a flat surface, and diffuse reflectance occurred according to the terrain conditions.

### 3.2. CRT (Calibrated Reference Tarp) BRDF (Bidirectional Reflectance Distribution Function) for ANIF (Anisotropy Factor)

As shown in Table 6, in the 1st CRT 3%, the standard deviations of the isometric ($k_{iso})$, volumetric ($k_{vol})$, and geometric ($k_{geo})$ coefficients were improved from ±1.58 to 0.82, from ±2.17 to 1.44, and from ±1.06 to 0.56, respectively. However, the CRT 3% and the other CRTs had completely different tendencies.

In the CRT 20%, 31%, and 46%, the ‘K’ ($k_{iso}$, $k_{vol}$, and $k_{geo}$) coefficient was improved from 1.02 ± 0.42 to 1.12 ± 0.04, from 0.42 ± 0.44 to 0.38 ± 0.12, and from 0.36 ± 0.67 to 0.06 ± 0.02, respectively, which was calculated using the ‘K’ coefficients’ average and standard deviation of each flight. Before the correction, the CRT 20%, 31%, and 46% ANIF had slightly different tendencies. However, after correction, the CRT 20%, 31%, and 46% ANIF had similar tendencies.

**Figure 6:**
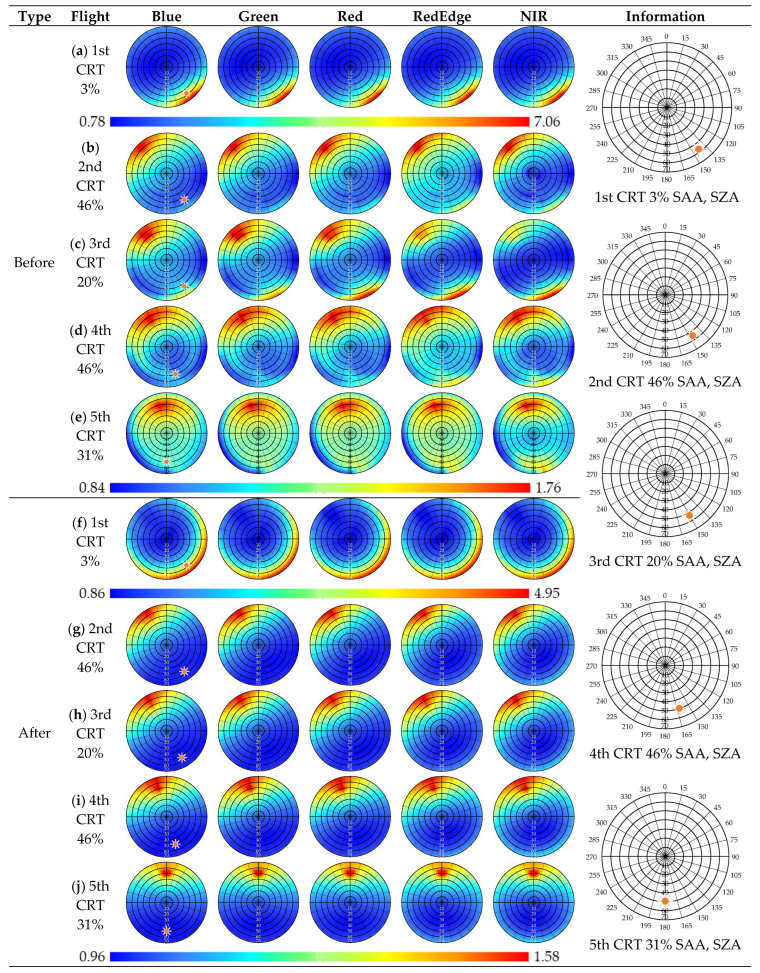
The ANIF results of the CRT (calibrated reference tarp) before and after by correction type.

In Figure 6, the ‘Before’ images are the ANIF using P4M irradiance based on the blue line in Figure 5b, and the ‘After’ images are the ANIF using ground irradiance based on the gray line in Figure 5b. In the ANIF before and after irradiance correction, we calculated the ‘K’ coefficient ($k_{iso}$, $k_{vol}$, and $k_{geo}$). In the 1st CRT 3%, a hotspot occurred at the SAA and SZA point. This result was quite different from the results of other CRTs.

In the 2nd CRT 46%, the weather was stable before the VZA 20° flight. However, the irradiance decreased from VZA 20°, and the reflectance became relatively low at VZA 10° to 20°, as shown in Figure 6b, due to the weather conditions.

In the 3rd CRT 20%, as the VZA increased, the reflectance also increased. The adjacency radiance was found at VZA 70° in RE and NIR. The results, shown in Figure 6h after correction using the ground irradiance, were stable. 

In the 4th CRT 46%, the images were affected by clouds and the results, shown in Figure 6d, seem to be relatively less stable than the 2nd CRT 46%. The results after correction with ground observation irradiance were stable, as shown in Figure 6i.

In the 5th CRT 31%, Figure 6j shows the results of calibration with ground irradiance. The influence of the clouds, which were present for the earliest 4 min of the flight, is illustrated in the results in Figure 5a, and these were less stable than the 2nd CRT 46% results.

In the results of calculating the ANIF, the reflectance hotspot occurred when SZA and SAA + 180° and VZA and VAA were similar in CRT 20%, 31%, and 46%. When the VZA was 60~70° in the RE and NIR, the high VZA reflectance was higher than the low VZA reflectance.

Table 7 shows the standard deviation of reflectance after P4M irradiance correction. According to the results of the 1st CRT 3% flight, the standard deviation of the check and BRDF image was ±0.3~0.6% and ±1.1~2.7%, respectively. In the 2nd CRT 46% flight, the standard deviation of the check and BRDF image was ±2.2~2.9% and ±1.8~2.5%, respectively. In the 3rd CRT 20%, the standard deviation of the check and BRDF image was ±2.5~4.1% and ±1.5~4.1%, respectively. In the 4th CRT 46%, the standard deviation of the check and BRDF image was ±0.9~2.1% and ±1.7~4.0%, respectively. In the 5th CRT 31%, the standard deviation of the check and BRDF image was ±0.9~2.6% and ±1.7~4.5%, respectively.

Generally, the uncertainty of the reflectance and the net radiation was 10% and 5%, respectively, for the BRDF in clear weather [35]. For the ground ASD with RCR, it was calculated within a range of 10% deviation from all CRT and bands, excluding some RE and NIR bands.

Therefore, from among the examples, no irradiance correction, irradiance correction of the P4M sunlight sensor, and ground irradiance correction of ASD with RCR, when there were no clouds and low water vapor weather such as in a desert, no irradiance correction, and the irradiance of the ASD with RCR were the appropriate methods. The irradiance of the P4M sunlight sensor was not an appropriate method in areas with weather changes and water vapor changes.

In Table 8, the CRT 3% ANIF was 0.99~1.02 ± 0.10~0.32. The CRT 20%, 31%, and 46% ANIF values for each band were 1.05~1.06 ± 0.05~0.07 (blue), 1.05~1.06 ± 0.05~0.06 (green), 1.04~1.05 ± 0.05~0.06 (red), 1.02 ± 0.05~0.06 (RE), and 1.06~1.07 ± 0.06~0.07 (NIR). In Table 8, considering that the reflectance of the CRT 46% observed in the field was 44.7~46.1%, the results calculated in the check image were either closely identical to or approximately 0.5% bigger than the nadir image. At the nadir, it was approximately 1.7~2.6 bigger than the field measured reflectance. CRT 20%, 31%, and 46% were similar, except for the CRT 3%.

## 4. Discussion

Table 9 shows the results for the correlation coefficients of the ANIF values for each flight. The CRT 3% had no correlation with CRT 20%, 31%, and 46%. In addition, when the CRT 20%, 31%, and 46% also did not perform a time match, the correlation coefficient was 0.792 to 0.996. However, the time difference between the second and fifth flights was approximately 01:51:14, and the correlation coefficient according to the time change decreased as the time difference increased.

However, when the time was matched with the solar meridian time (12:15:28) at 29 October 2021, the solar meridian time SAA and SZA was 180.0° and 49.3°, respectively. The CRT 3% had no correlation with CRT 20%, 31%, and 46%. The correlation coefficients were similar at 0.992 to 0.998 in CRT 20%, 31%, and 46%.

At CRT 3%, the irradiance between the air (P4M sunlight sensor) and on the ground (ASD with RCR) was small or similar, but, at CRT 20%, 31%, and 46%, the ANIF correlation was very high. This was because the CRT 3% was greatly affected by the adjacency effect, and CRT 20%, 31%, and 46% were affected by the weather conditions. Although the improvement effect was shown in all bands, the improvement effect by correction was relatively low for the SZA and CRT in the RE and NIR bands. In VIS, the absolute amount of change in reflectance was stable, but the relative amount of change occurred largely due to the influence of VAA and SZA.

Incident energy is largely divided into direct and indirect energy. Direct energy is the energy provided directly from the Sun, and all energy is called the adjacency effect or diffused energy.

This is because, although the adjacency or diffuse effect is low at 400 to 700 nm (B, G, R), it is about twice as much affected at the 730 nm (RE) band, and 3~4 times more affected at the 840 nm (NIR) band [39]. The adjacency effect occurs with oxygen, water vapor adsorption, and Rayleigh and aerosol scattering. Water vapor absorption is affected at 710–735 nm and 805–840 nm, and oxygen is affected at 755–770 nm. Therefore, in the RE band, it is directly affected by water vapor and indirectly affected by oxygen. In the NIR band, many errors appear due to the influence of water vapor.

The adjacency effect is a phenomenon that changes the ground radiance through; for example, increasing the top of atmosphere (TOA) radiance of the dark pixels and decreasing the radiance of the bright pixels [40].

The direct radiance and adjacency radiance in each CRT from the fieldwork site were analyzed using ATCOR [41]. The input water vapor and visibility parameters were 1.0 g/m^3^ and 20 km, respectively, and the range of the adjacency effect was 300 m. The direct radiance ratio was $\;L^{su}{}_{\lambda }/L^{adj}{}_{\lambda }$ for each CRT. 

The $L^{su}{}_{\lambda }$ is the surface-reflected radiation at the target, and $L^{adj}{}_{\lambda }$ is the adjacency radiance at the target surrounding zone and the surface structure reflected in the skylight path of the radiance [42].

As shown in Table 10, the ratio of adjacent light to direct light observed by the UAV was 1.059~2.405 (CRT 3%), 0.640~0.681 (CRT 20% in VIS), 1.133 (CRT 20% in RE and NIR), 0.492~0.813 (CRT 31%), and 0.333~0.522 (CRT 46%).

In the CRT 3%, the main light source was considered to be an adjacent light because the diffused energy was higher than the direct energy at a lower SZA. Therefore, in general, the location of the hotspot was at SAA + 180°, but the CRT 3% result indicated that the hotspot was located at SAA (in Figure 6f). 

In CRT 20%, 31%, and 46%, the main light source of the VIS band was expected to be direct light, but the main light source of the RE and NIR bands was concluded to be adjacent light. Therefore, we established that it had high reflectance, even after being processed at a high VZA that was greatly affected by the diffuse light such as the RE and NIR bands in Figure 6g–j 

To analyze the effects of reflectance, the ANIF results of the check image in all bands were 1.01 ± 0.19 (CRT 3%), 1.05 ± 0.06 (CRT 20%), 1.05 ± 0.06 (CRT 31%), 1.05 ± 0.05 (CRT 1st 46%), and 1.05 ± 0.06 (CRT 2nd 46%), as shown in Table 8. Adjacency light was less in the upper 20% reflectance CRT by SZA and reflectance, but adjacent light was large in the CRT 3%.

The environment or adjacency effect can be clarified by the reflection condition. Most of the energy comes from the target. However, due to atmospheric scattering or absorption, the diffused energy in the air is reflected on the adjacent surrounding target. Therefore, the adjacency effect is the spectral disturbance of the target due to the factors around it [43].

The adjacency effects are known to be lower in open-ocean environments [44]. However, the adjacency effect can increase at low albedo targets. Therefore, in CRT 3%, the adjacency effect was dominant, so the result was different.

The reflectance hotspot was at SAA + 180° and SZA, and the RE and NIR bands were more affected than the VIS bands. In addition, cloudy weather results are more likely to be wrong compared to results produced by clear weather. As the SZA increases, the $k_{geo}$,$\;k_{vol}$ also increase [45]. In this study, the observed values established the same tendency, SAA + 180°, and the highest point appeared in the SZA.

According to [46], as the SZA increased at a low reflectance (3~4%), it changed very rapidly with an estimate of up to 10 times. As the SZA increased in the gray reference target (15~17%), it increased approximately 2–3 times. In this study, considering that the SZA was 49~58°, the same phenomenon occurred in the high VZA area due to the adjacent light effect at the low SZA.

If the experiment had been conducted in the deserts with no clouds and very little impact produced by water vapor, then we would simply need to consider the SAA and SZA conditions. However, there were few cases of no clouds and low water vapor (0.1~0.5 g/m^3^) in South Korea.

Although the adjacency effect was calculated to be highly influenced by the RE and NIR bands, with the direct radiance, the VIS value was larger and the value in the RE and NIR band was lower.

The result of CRT 3% was different from the other CRTs. This phenomenon occurred at all bands of CRT 3% and partially occurred at the NIR band of CRT 20% and occurred due to the influence of $L^{adj}{}_{\lambda }$, which was 1.5 to 2 times more in the RE and NIR bands.

The reflectance error in the NIR band was larger than in the VIS band under perfect conditions (humidity and temperature-controlled laboratory).

## 5. Conclusions

This study aimed to determine whether a UAV-based goniometer, which has an effect similar to that of a classical goniometer, was useful. A multispectral camera with a controllable gimbal installed on a UAV can be operated in the hemisphere. In addition, the BRDF and anisotropic reflection using the CRT were evaluated. If a goniometer is operated together with a spectrometer in a laboratory, outstanding results can be obtained. 

The possibility of the field utilization of the equipment is determined by the mobility, price, accuracy, speed, and ability to solve expected problems. In this study, the equipment used was less mobile than traditional equipment, so it was appropriate. The price was approximately USD 10,000, which was more reasonable than that of the existing equipment. Accuracy was relatively low, but accuracy was expected at a reasonable level. The expected problems were that the accuracy would be reduced due to weather changes (cloud, atmospheric conditions), and the accuracy of the UAV mounted sunlight sensor would be greatly influenced by the flight attitude. To solve these problems, in this study, the ground observation data were used instead of UAV observation data, and most results resolved the problem.

Irradiance was measured in two ways: a sunlight sensor mounted on a P4M and a spectroradiometer with RCR. The P4M obtained unstable irradiance data, and the RCR installed on the ground obtained relatively stable data. The P4M was operated while changing its attitude. After correcting the nadir and check images with the RCR irradiance data (ground irradiance), the reflectance of the CRT was similar for each band. In addition, the reflectance at each CRT of the image before and after correction with ground irradiance data, and the reflectance values at each CRT derived by simulation through the BRDF model were calculated based on the BRDF image and the check image. The after correction value was similar to the simulation result.

When the ‘K’ values were calculated using images before and after correction with ground irradiance, the standard deviation of $k_{iso}$, $k_{vol,}$ and $k_{geo}$ was lower in the image after correction than before correction. At this time, the results calculated in CRT 3% and the others showed different tendencies. Comparing the results of correction with the P4M and the ground irradiance data, the results of correction with ground irradiance were much more stable. In addition, in general, hotspots with high ANIF results appeared at the same zenith angle as the SZA and at an azimuth angle of SAA + 180°, but this was not the case in CRT 3%. The standard deviation between the reflectance of each CRT in the image corrected with the P4M irradiance data and in each CRT derived by simulation through the BRDF model was calculated based on the BRDF image and the check image. The standard deviation values after correction were not similar to the simulation results.

Under the same experimental conditions, the ANIF results should be similar to each other, regardless of the reflectance of the CRT. Each flight was operated at different solar positions (SZA, SAA), and when these solar conditions were equal, the ANIF results were similar. In addition, as a result of checking how much direct light and indirect light affect each CRT and band, it was found that the lower the CRT reflectance and the longer the band wavelength, the greater the influence of indirect light.

In future, better results are expected if direct irradiance adapters (DIAs) and RCRs are used simultaneously to separate direct and diffuse irradiance. In addition, it is necessary to sufficiently study the object and irradiance of the surrounding representative objects that have a great influence on low reflectance targets.

## Figures and Tables

**Table 1:** P4 Multispectral (P4M) camera setting for BRDF (bidirectional reflectance distribution function) measuring (VAA: vehicle azimuth angle, VZA: vehicle zenith angle, pitch interval: 10°, and yaw interval: 15~30°, Unit: °, ea).

Flight Type	Information								
For hemisphere Radius: 20 m	VZA	70°	60°	50°	40°	30°	20°	10°	Nadir
	VAA interval	15°	15°	15°	15°	15°	20°	30°	-
	Counts of photo	24	24	24	24	24	18	12	1
For accuracy assessment	VAA	78°		168°		258°		348°	
	VZA interval	3°		3°		3°		3°	
	Counts of photo	11		11		11		11	

**Table 2:** Time, SZA (sun zenith angle), and SAA (sun azimuth angle) for each flight and CRT (calibrated reference tarp) type.

Flight	Start	End	Flight Time	SAA	SZA	CRT Type
1st CRT 3%	10:03:21	10:14:22	11:01	142.6	57.8	3%
2nd CRT 46%	10:17:11	10:28:14	11:03	146.2	56.1	46%
3rd CRT 23%	10:31:45	10:42:46	11:01	149.9	54.6	23%
4th CRT 46%	11:11:29	11:22:31	11:02	161.3	52.3	46%
5th CRT 31%	12:08:23	12:19:30	11:07	179.2	49.3	31%

**Table 3:** The reflectance average and standard deviation in each flight and CRT (calibrated reference tarp) after correction irradiance using ASD with RCR (remote cosine receptors) (Avg: average, Std: standard deviation, unit: %).

Flight	Nadir (1 ea per Band)					Check (44 ea per Band)											
	Avg					Avg						Std					
	B	G	R	RE	N	B	G	R	RE	N	Tot	B	G	R	RE	N	Total
1st	2.0	2.2	2.1	1.8	1.2	2.3	2.4	2.4	2.2	1.7	2.2	0.2	0.3	0.3	0.4	0.5	0.5
2nd	45.6	46.4	46.4	46.6	42.4	46.0	46.9	46.9	46.9	43.4	46.0	2.9	2.9	2.9	1.5	3.6	3.2
3rd	19.1	17.7	15.8	12.8	10.8	19.4	18.1	16.3	13.4	11.7	15.8	1.4	1.2	1.1	1.2	1.5	3.1
4th	45.0	45.4	45.0	44.4	49.7	45.9	46.2	45.8	44.8	50.8	46.7	3.0	3.0	2.8	1.4	3.6	3.5
5th	25.9	25.6	22.5	17.4	20.7	26.4	26.0	22.9	17.6	21.4	22.9	1.8	1.6	1.4	0.9	2.0	3.6

**Table 4:** The reflectance difference deviation before and after correction (unit: %).

BRDF (150 ea per Band)		No Irradiance Correction					After Irradiance Correction				
Check (44 ea per Band)		VIS			RE, NIR		VIS			RE, NIR	
		B	G	R	RE	NIR	B	G	R	RE	NIR
In BRDF Image (VZA: 0~70°)	1st CRT 3%	0.9	1.2	1.4	1.7	2.6	1.1	1.3	1.5	1.9	2.6
	2nd CRT 46%	2.8	3.3	3.6	3.3	4.9	1.8	1.9	2.1	1.8	3.5
	3rd CRT 20%	1.7	1.9	2.2	2.7	4.2	1.5	1.7	2.0	2.5	4.1
	4th CRT 46%	5.9	7.0	7.3	7.8	9.0	2.7	2.3	2.6	1.9	4.3
	5th CRT 31%	3.5	3.9	3.9	3.1	5.3	2.2	2.1	2.2	1.7	4.5
In Check image (VZA: 0~30°)	1st CRT 3%	0.3	0.3	0.3	0.4	0.6	0.3	0.3	0.4	0.4	0.5
	2nd CRT 46%	4.7	5.6	6.1	5.5	8.2	2.6	3.0	3.7	2.2	5.9
	3rd CRT 20%	3.0	3.4	3.3	2.7	2.6	3.0	2.5	2.5	2.9	4.1
	4th CRT 46%	1.4	1.5	1.8	1.7	1.8	1.1	1.0	1.3	0.9	2.0
	5th CRT 31%	1.8	1.9	1.9	1.5	2.8	1.1	1.2	1.2	0.9	2.6

**Table 5:** In the CRT (calibrated reference tarp), reflectance uncertainty after correction irradiance using ASD with RCR (remote cosine receptors) by ground condition (unit: %).

	In Check Image					In BRDF Image				
	B	G	R	RE	NIR	B	G	R	RE	NIR
1st CRT 3%	0.02	0.02	0.02	0.03	0.04	0.02	0.03	0.03	0.04	0.05
2nd CRT 46%	1.84	2.07	2.30	2.76	3.31	1.84	2.12	2.35	2.67	3.50
3rd CRT 20%	0.36	0.38	0.40	0.44	0.60	0.36	0.40	0.42	0.46	0.64
4th CRT 46%	1.61	1.75	1.98	2.21	3.13	1.61	1.79	1.93	2.30	3.08
5th CRT 31%	0.81	0.87	0.93	1.05	1.64	0.74	0.81	0.87	0.96	1.58

**Table 6:** ‘K’ (isotropic $\left(k_{iso}\right)$, volumetric $\left(k_{vol}\right)$, and geometric $\left(k_{geo}\right)$ scattering effect) coefficients of ANIF (anisotropy factor) after correction.

**Flight**	Coefficient	No Irradiance Correction					After Irradiance Correction				
		B	G	R	RE	NIR	B	G	R	RE	NIR
1st CRT 3%	$k_{iso}$	0.060	−0.012	−0.164	2.563	−1.845	0.040	−0.040	−0.216	−0.627	−1.945
	$k_{vol}$	1.695	1.713	1.893	−0.991	5.133	1.960	1.966	2.189	2.951	5.361
	$k_{geo}$	−0.587	−0.645	−0.742	1.011	−1.960	−0.668	−0.729	−0.844	−1.126	−2.026
2nd CRT 46%	$k_{iso}$	1.052	1.070	1.060	0.260	1.038	1.163	1.158	1.133	1.050	1.118
	$k_{vol}$	0.410	0.442	0.462	0.030	0.788	0.325	0.330	0.325	0.189	0.502
	$k_{geo}$	0.086	0.080	0.071	0.998	0.048	0.085	0.079	0.071	0.027	0.046
3rd CRT 20%	$k_{iso}$	1.150	1.129	1.067	1.081	0.806	1.162	1.156	1.131	1.048	1.115
	$k_{vol}$	0.509	0.598	0.718	−0.052	1.883	0.358	0.363	0.357	0.206	0.544
	$k_{geo}$	0.067	0.033	−0.003	1.026	−0.191	0.087	0.081	0.072	0.027	0.045
4th CRT 46%	$k_{iso}$	1.138	1.132	1.106	0.266	1.088	1.156	1.150	1.124	1.044	1.106
	$k_{vol}$	0.451	0.459	0.449	0.020	0.657	0.421	0.428	0.419	0.236	0.626
	$k_{geo}$	0.083	0.078	0.068	1.495	0.036	0.087	0.082	0.072	0.024	0.040
5th CRT 31%	$k_{iso}$	1.508	1.554	1.534	−0.058	1.388	1.147	1.143	1.116	1.039	1.098
	$k_{vol}$	0.014	−0.063	−0.034	0.160	0.503	0.392	0.402	0.392	0.221	0.596
	$k_{geo}$	0.192	0.196	0.185	2.563	0.119	0.076	0.072	0.061	0.017	0.026

**Table 7:** The reflectance standard deviation in the check image and BRDF image after the reflectance of each CRT was corrected with P4M irradiance (unit: %).

	In Check Image					In BRDF Image					Total	
	B	G	R	RE	NIR	B	G	R	RE	NIR	Check	BRDF
1st CRT 3%	0.3	0.3	0.4	0.4	0.6	1.1	1.3	1.6	1.9	2.7	0.4	1.7
2nd CRT 46%	2.6	3.0	3.7	2.2	5.9	1.8	1.9	2.2	1.8	3.5	3.5	2.2
3rd CRT 20%	3.0	2.6	2.5	2.9	4.1	1.5	1.7	2.0	2.5	4.1	3.0	2.4
4th CRT 46%	1.1	1.0	1.3	0.9	2.1	2.5	2.1	2.4	1.7	4.0	1.2	2.6
5th CRT 31%	1.1	1.2	1.2	0.9	2.6	2.2	2.1	2.2	1.7	4.5	1.4	2.5

**Table 8:** The ANIF (anisotropy factor) average and standard deviation in check image after correction.

Band	Average						Standard Deviation					
	B	G	R	RE	NIR	Total	B	G	R	RE	NIR	Total
1st CRT 3%	1.07	1.09	1.09	1.12	1.22	1.12	0.18	0.20	0.24	0.32	0.54	0.33
2nd CRT 46%	0.95	0.96	0.96	0.98	0.94	0.96	0.08	0.08	0.09	0.05	0.14	0.09
3rd CRT 20%	1.12	1.12	1.10	1.13	1.22	1.14	0.12	0.12	0.14	0.19	0.32	0.20
4th CRT 46%	1.00	1.02	1.01	1.01	1.02	1.01	0.06	0.05	0.05	0.02	0.06	0.05
5th CRT 31%	1.02	1.04	1.03	1.01	1.09	1.04	0.09	0.09	0.09	0.08	0.19	0.12

**Table 9:** The correlation of each flight’s ANIF (anisotropy factor) after correction by SAA and SZA.

Type		Difference SAA, SZA (H:M:S)					Same SAA, SZA				
Time Difference		0	0:13:51	0:14:33	0:39:45	0:56:56	0				
CRT Type	Flight	1st	2nd	3rd	4th	5th	1st	2nd	3rd	4th	5th
CRT 3%	1st	1.000	−0.073	−0.070	−0.049	0.023	1.000	−0.084	−0.063	−0.012	0.034
CRT 46%	2nd	−0.073	1.000	0.996	0.948	0.792	−0.084	1.000	1.000	0.996	0.992
CRT 20%	3rd	−0.070	0.996	1.000	0.969	0.830	−0.063	1.000	1.000	0.998	0.994
CRT 46%	4th	−0.049	0.948	0.969	1.000	0.921	−0.012	0.996	0.998	1.000	0.998
CRT 31%	5th	0.023	0.792	0.830	0.921	1.000	0.034	0.992	0.994	0.998	1.000

**Table 10:** The field-measured reflectance on CRT, and the ratio of the direct and adjacency effects by ATCOR.

Index	Reflectance by ASD				Adjacency and Direct Ratio			
Band	B	G	R	RE, N	B	G	R	RE, N
CRT 3%	2.7	2.7	2.8	3.0	1.059	1.201	1.360	2.405
CRT 20%	20.9	19.7	18.4	17.1	0.681	0.663	0.640	1.133
CRT 31%	31.5	30.5	29.3	28.2	0.579	0.536	0.492	0.813
CRT 46%	46.1	45.4	44.7	45.6	0.442	0.387	0.333	0.522

## Data Availability

Data sharing not applicable.
